# Role of Brain Liver X Receptor in Parkinson’s Disease: Hidden Treasure and Emerging Opportunities

**DOI:** 10.1007/s12035-023-03561-y

**Published:** 2023-08-22

**Authors:** Saud A. Alnaaim, Hayder M. Al-Kuraishy, Athanasios Alexiou, Marios Papadakis, Hebatallah M. Saad, Gaber El-Saber Batiha

**Affiliations:** 1 Clinical Neurosciences Department, College of Medicine, King Faisal University, Hofuf, Saudi Arabia; 2 Department of Clinical Pharmacology and Therapeutic Medicine, College of Medicine, ALmustansiriyiah University, Baghdad, 14132 Iraq; 3 Department of Science and Engineering, Novel Global Community Educational Foundation, Hebersham, NSW 2770 Australia; 4 AFNP Med, 1030 Wien, Austria; 5 Department of Surgery II, University Hospital Witten-Herdecke, Heusnerstrasse 40, University of Witten-Herdecke, 42283 Wuppertal, Germany; 6 Department of Pathology, Faculty of Veterinary Medicine, Matrouh University, Marsa Matruh, 51744 Egypt; 7 Department of Pharmacology and Therapeutics, Faculty of Veterinary Medicine, Damanhour University, Damanhour, AlBeheira 22511 Egypt

**Keywords:** Parkinson’s disease, Liver X receptor, Neurodegenerative diseases

## Abstract

Parkinson’s disease (PD) is a neurodegenerative disease due to the degeneration of dopaminergic neurons (DNs) in the substantia nigra (SN). The liver X receptor (LXR) is involved in different neurodegenerative diseases. Therefore, the objective of the present review was to clarify the possible role of LXR in PD neuropathology. LXRs are the most common nuclear receptors of transcription factors that regulate cholesterol metabolism and have pleiotropic effects, including anti-inflammatory effects and reducing intracellular cholesterol accumulation. LXRs are highly expressed in the adult brain and act as endogenous sensors for intracellular cholesterol. LXRs have neuroprotective effects against the development of neuroinflammation in different neurodegenerative diseases by inhibiting the expression of pro-inflammatory cytokines. LXRs play an essential role in mitigating PD neuropathology by reducing the expression of inflammatory signaling pathways, neuroinflammation, oxidative stress, mitochondrial dysfunction, and enhancement of BDNF signaling.

In conclusion, LXRs, through regulating brain cholesterol homeostasis, may be effectual in PD. Also, inhibition of node-like receptor pyrin 3 (NLRP3) inflammasome and nuclear factor kappa B (NF-κB) by LXRs could effectively prevent neuroinflammation in PD. Taken together, LXRs play a crucial role in PD neuropathology by inhibiting neuroinflammation and associated degeneration of DNs.

## Introduction

Parkinson’s disease (PD) is the second most common neurodegenerative disease next to Alzheimer’s (AD). PD was first recognized by James Parkinson in 1817 as shaking palsy [1]. The incidence of PD is about 1% in the general population, which increased to 2% in subjects over sixty; however, this percentage is highly augmented above the age of eighty [2]. Therefore, PD is regarded as an age-related disorder due to age-induced progressive neuronal loss [3]. PD is developing due to the progressive degeneration of dopaminergic neurons (DNs) in the substantia nigra (SN) of the midbrain [4]. The causes of PD are related to the alteration of genetic and epigenetic variants [5]. Environmental toxins and stress are involved in triggering PD neuropathology [6]. The pathognomonic feature of PD is the deposition of α-synuclein and aggregation of Lewy bodies [7]. In fact, α-synucleinopathy is not restricted to the SN but affects the entire central nervous system (CNS), including the autonomic nervous system which could be the initial site in the development and progression of PD [8]. The latent period between PD neuropathology and symptomatic PD may be many years [9]. Cardinal motor symptoms of PD include resting tremors, rigidity, bradykinesia, shuffling gait, and instability [10]. Besides, various non-motor symptoms, including sleep disturbances, apathy, anxiety, depression, neuropsychiatric disorders, dementia, autonomic dysfunction, sensory abnormality, and cognitive deficits, are developed [11]. Remarkably, non-motor symptoms may be the initial feature as they develop several years before classic motor symptoms and may be misdiagnosed with psychiatric disorders [12]. Various cellular disorders, including inflammation, autophagy, mitochondrial dysfunction, endoplasmic reticulum (ER) stress, and microgliosis, are involved in PD neuropathology [13, 14].

PD is commonly associated with cardiometabolic disturbances, directly and indirectly affecting PD neuropathology [15]. It has been shown that metabolic syndrome and its components are linked with the development of PD [16]. A nationwide cohort study involving 17,163,560 subjects aged > 40 years in South Korea found that components of metabolic syndrome like hypertriglyceridemia and low-density lipoprotein (LDL) were associated with PD risk [17]. The liver X receptor (LXR) involves lipid homeostasis and different cardiometabolic disorders [18]. LXR is also shown to engage with neurodegenerative diseases like AD [18, 19]. Therefore, the objective of the present review was to clarify the possible role of LXR in PD neuropathology.

## Liver X Receptor

Nuclear receptors are the master body homeostasis regulator that controls most biological processes [20]. LXRs are the most common nuclear receptors of transcription factors that regulate cholesterol metabolism and are involved in different pathologies, including atherosclerosis, cancer, neurodegenerative diseases, chronic inflammation, and autoimmunity [20]. LXRs are closely related to other types of receptors like peroxisome proliferators activated receptors (PPARs), farnesoid X receptor (FXR), and retinoid X receptor (RXR) [21]. LXRs are of two types; LXRα was discovered in 1994 and initially named an RLD-1 receptor, though LXRβ was discovered separately simultaneously and was known as RIP-15. Genes of LXRα and LXRβ were identified to be located on chromosome 11p11.2 and chromosome 19q13.3, respectively [22]. LXRs are expressed in different tissues with considerable overlap. LXRβ is expressed in all tissues, so-called ubiquitous receptors (UR) though LXRs are mainly expressed in the liver, kidney, adipose tissues, intestine, spleen, lung, and macrophages [23]. Expression differences of these receptors suggest a different physiological role. LXRs regulate immune response and mediate anti-inflammatory effects by promoting the expression of inflammatory genes and mediators in response to different microbial infections [24, 25]. LXRs inhibit dendritic cells and macrophage activity as well as migration and proliferation of lymphocytes [24, 25].

Inflammation and cholesterol homeostasis are closely related to regulating inflammation and immune response [24, 25]. Besides, LXRs have a crucial anti-tumor role and regulate cancer biology by regulating natural killer T cell responses [26]. LXRs have pleiotropic effects on the tumor microenvironment [26]. Of note, LXRs are essential in cholesterol metabolism, and cholesterol derivatives, including oxysterols and desmosterol, act as LXRs activators [27]. Activation of LXRs provokes LXR heterodimerization with RXR leading to activation of LXR response element with subsequent gene activation involved in glucose and lipid metabolism [28, 29]. LXRs regulate abnormal intracellular sterol by activating the expression of ATP-binding cassette (ABC), carbohydrate response element binding protein (ChREBP), and sterol regulatory element binding protein 1c (SREBP1c) that control lipogenesis [30]. Furthermore, LXRs regulate low-density lipoprotein (LDL) expression and increase uptake of cholesterol and LDL particles through induction expression of LDL receptor (LDLR) and inducible degrader of LDLR (IDOL) [31]. Of interest, LXRs are regarded as a potential link between the immune system and cholesterol homeostasis [32].

It has been observed that LXRs are involved in the pathogenesis of atherosclerosis through the induction of hyperlipidemia. LXRα knockout mice develop fatty liver when fed on a high-fat diet, though LXRβ knockout mice did not develop lipid disorders when fed on a high-fat diet [33, 34]. These verdicts suggested a differential role of LXRs on lipid metabolism. As well, LXRs contribute to the regulation of brain cholesterol metabolism [19]. LXRs knockout mice develop neurodegeneration due to cholesterol-induced synaptic dysfunction and neuronal loss [35]. Of interest, adiponectin attenuates the development of neurodegeneration through the activation of LXRs [36]. In general, LXR agonists can potentially manage AD, inflammation, diabetes, and atherosclerosis [37].

**Fig. 1:**
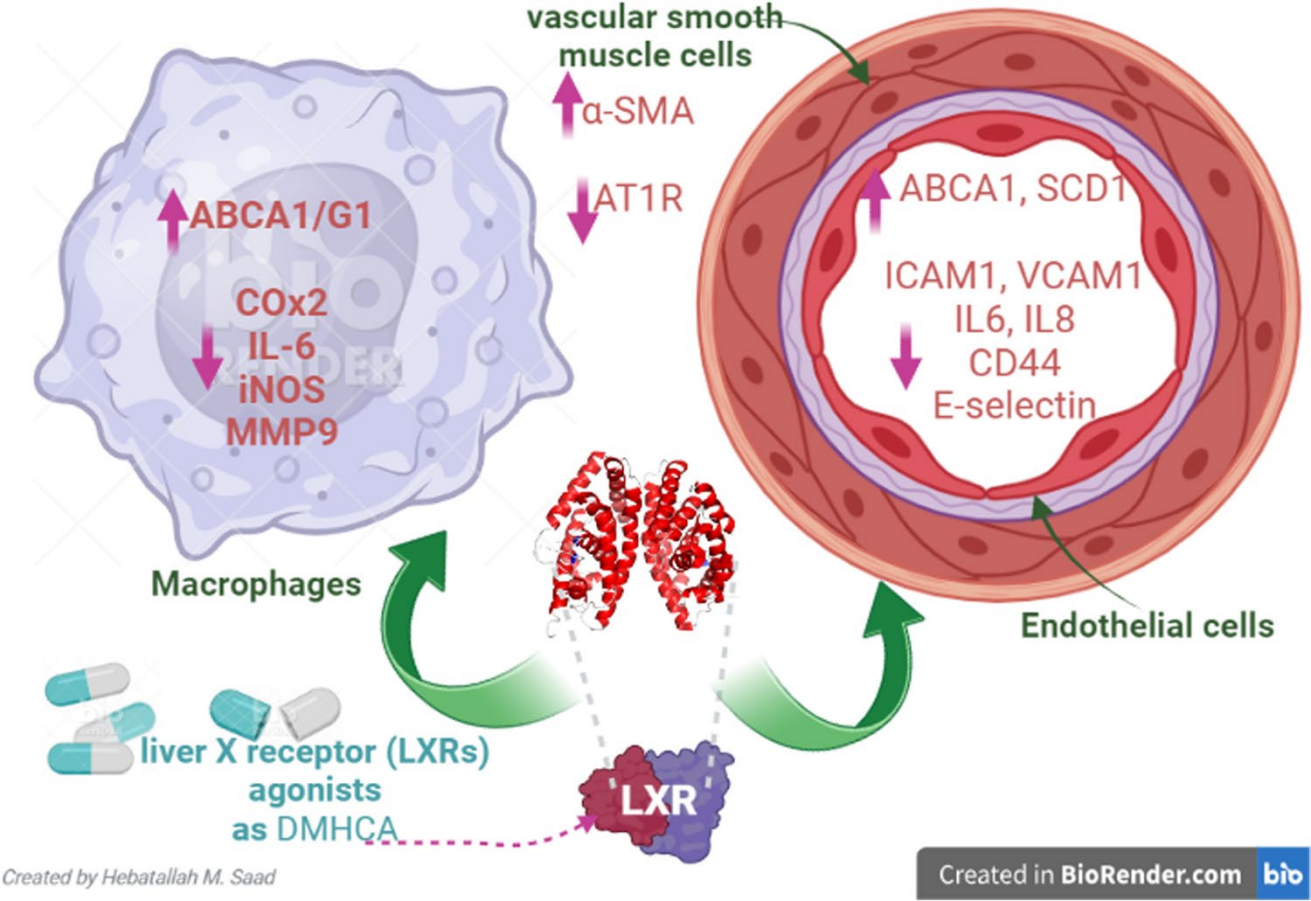
The potential effect of liver X receptor (LXRs) agonists: LXR agonists advance vascular smooth muscle cells by increasing the expression of alpha-smooth muscle actin (α-SMA) and decreasing the expression of angiotensin 1 receptor (AT1R). LXR agonists encourage the functional capacity of endothelial cells by increasing the expression of ATP-binding cassette A1 (ABCA1) and STEAROYL-CoA desaturase-1 (SCD-1) and reducing the expression of adhesion molecule and pro-inflammatory cytokines. In macrophages, LXR agonists upsurge expression of ABCA1 and mer tyrosine kinase receptor with reduced expression of IL-6, cyclooxygenase 2 (COX-2), inducible nitric oxide synthase (iNOS), and matrix metalloproteinase 9 (MMP-9)

Furthermore, LXRs improve insulin sensitivity and attenuate obesity-induced insulin resistance by controlling gene expression involved in glucose metabolism in the liver and adipose tissues [38]. Aberrant expression of LXRs in the macrophages is developed under the effect of oxidized cholesterol like 7-ketocholesterol leading to atherosclerosis [39, 40]. Thus, inhibition of 7-ketocholesterol could be a possible pathway in treating atherosclerosis [41]. Notably, LXR agonists may lead to increase production of triglyceride and VLDL [42]. Besides, hyperglycemia in diabetes induces the expression LXRs, causing hypertriglyceridemia [43]. This undesirable effect of LXRs agonists is due to the competition of LXRs with PPARα on heterodimerization with a limited pool of RXR [43]. However, potent and selective LXR agonists like N, N-dimethyl-3β-hydroxy-cholenamide (DMHCA) decrease atherosclerosis in ApoE-deficient mice without the development of liver steatosis and hypertriglyceridemia [37]. LXR agonists significantly regulate inflammatory mediators in endothelial cells, vascular smooth muscle cells, and macrophages [44]. LXR agonists improve vascular smooth muscle cells by increasing the expression of alpha-smooth muscle actin (α-SMA) and reducing the expression of angiotensin 1 receptor (AT1R) [44]. Furthermore, LXR agonists promote the functional capacity of endothelial cells by increasing the expression of ABCA1 and STEAROYL-CoA desaturase-1 (SCD-1) and reducing the expression of adhesion molecules and pro-inflammatory cytokines. In macrophages, LXR agonists increase the expression of ABCA1 and mer tyrosine kinase receptor with reduced expression of IL-6, cyclooxygenase 2 (COX-2), inducible nitric oxide synthase (iNOS), and matrix metalloproteinase 9 (MMP-9) [44] (Fig. 1).

**Fig. 2:**
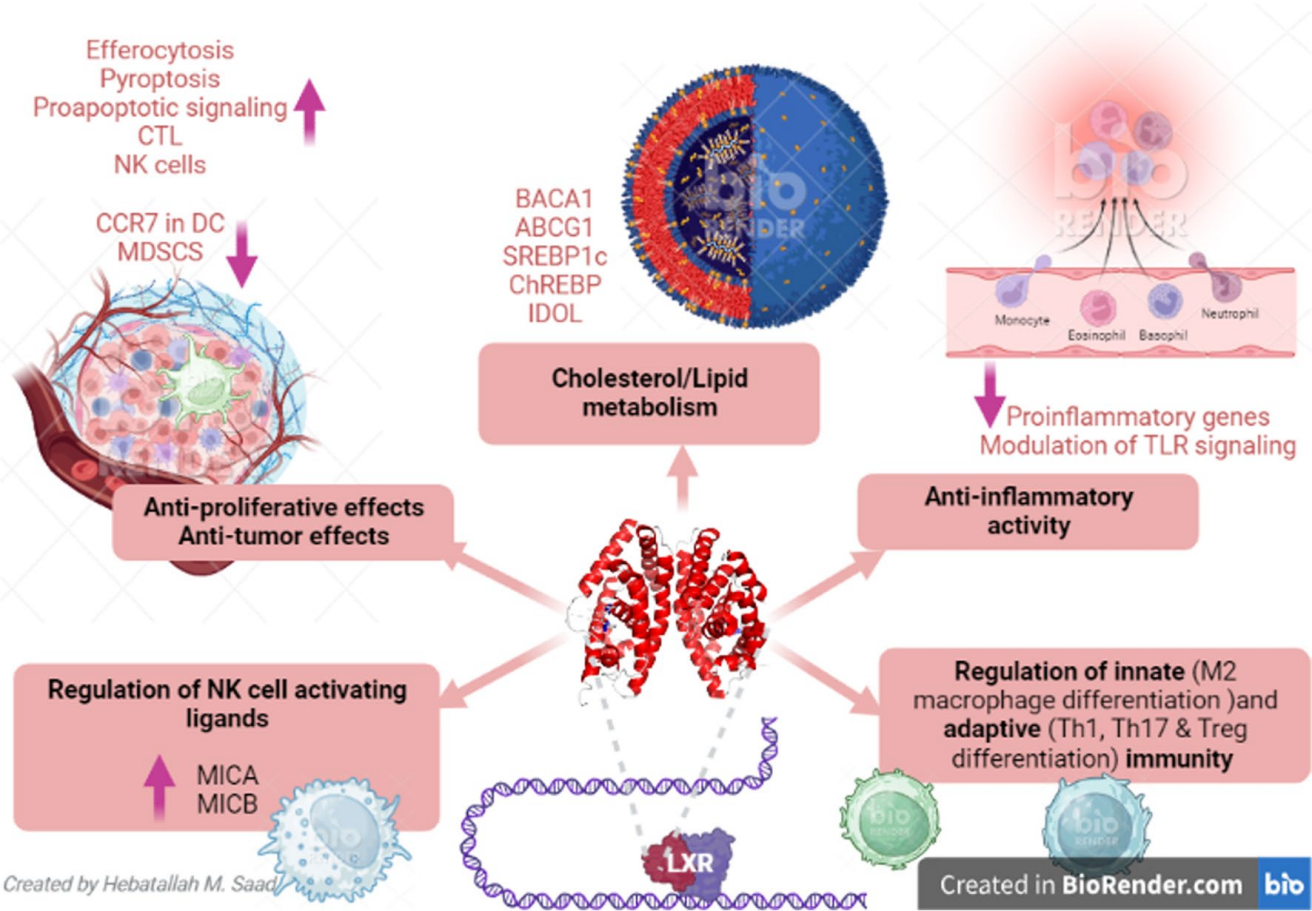
Potential functional role of LXRs: LXR agonists could be a promising therapeutic target in cancer, prostatic carcinoma, Goodpasture disease (GBM), rheumatoid arthritis (RA), systemic lupus erythematosus (SLE), and irritable bowel disease (IBD). LXR agonists produce their effects by pleiotropic effects including anti-inflammatory effects, reduced intracellular cholesterol accumulation, immune regulation, anti-proliferative effects, anti-tumor effects, and prevention of the development of endoplasmic reticulum stress

Taken together, LXRs have pleiotropic effects, including anti-inflammatory effects, reduced intracellular cholesterol accumulation, immune regulation, anti-proliferative effects, and anti-tumor effects, and prevent development of endoplasmic reticulum stress (Fig. 2).

## Brain Cholesterol and LXRs

The brain has an advanced cholesterol concentration compared to any other organ in the body; it has 25% of the total cholesterol in the body [45]. Brain cholesterol chiefly exists as an unesterified form [46]. Blood cholesterol cannot cross blood brain barrier (BBB); hence, brain *de novo* cholesterol biosynthesis is the foremost source of brain cholesterol [47]. Though, evidence from preclinical studies demonstrated that lipoprotein-bound cholesterol can cross BBB [48]. Particularly, scavenger receptor type B1 (SR-B1) expressed on cerebral capillary endothelial cells plays a vital role in cholesterol uptake from LDL and HDL [48]. Additionally, brain endothelial cells can uptake LDL via LDLR [49]. These pathways donate to a minor route for cholesterol transport from peripheral circulation into the brain.

**Fig. 3:**
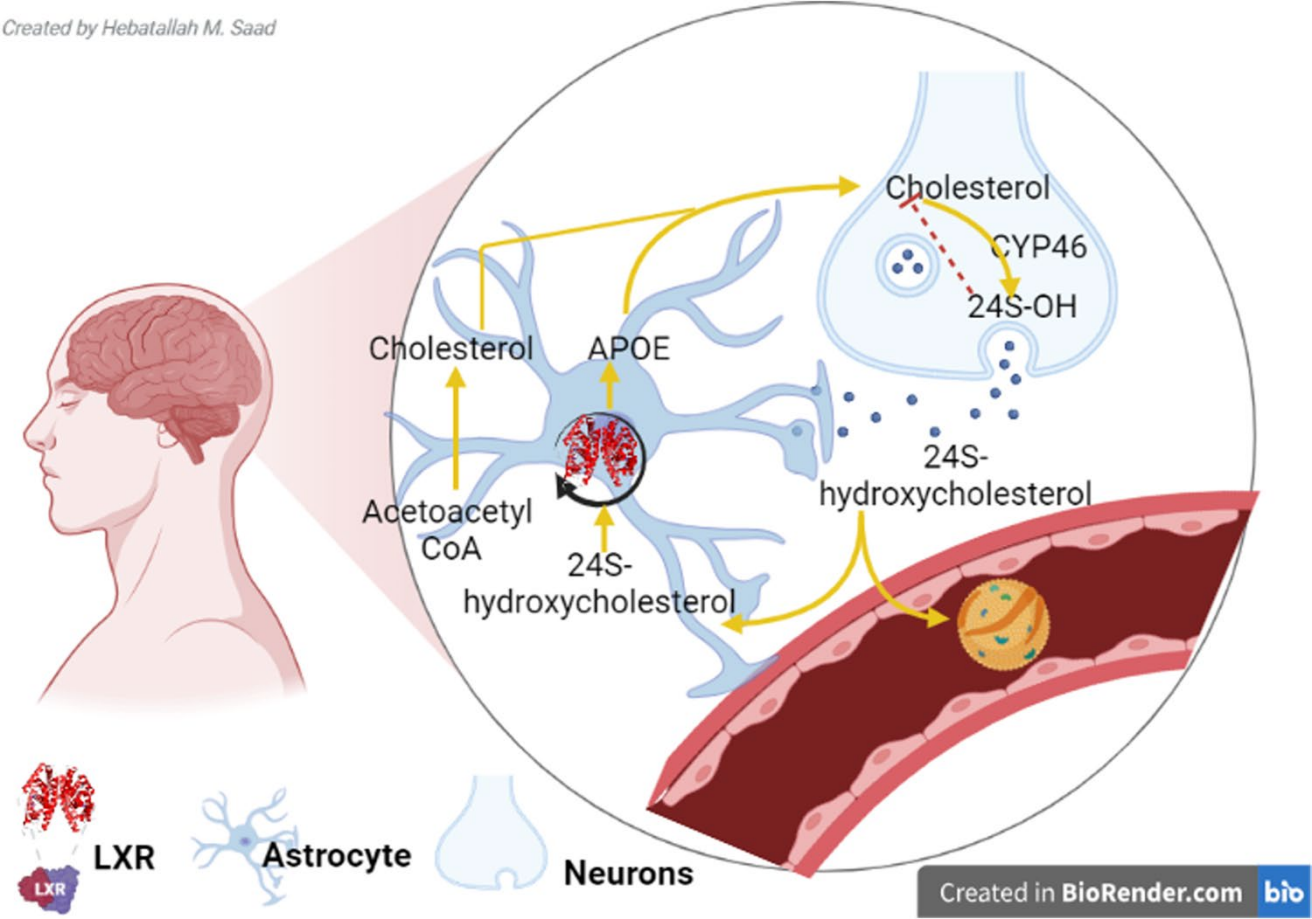
Brain cholesterol homeostasis: Astrocytes synthesize cholesterol which is transported with the assistance of ApoE via ATP-binding cassette (ABCA1) to neurons. Cholesterol in the neurons is metabolized to 24S-hydroxycholesterol (24S-OH), which is transported to astrocyte and inhibit cholesterol biosynthesis. Some of 24S-OH are regulated by the liver X receptor (LXR) and excreted via ABCA1 to synthesize cholesterol in the neurons. However, the other part of 24S-OH is eliminated from the systemic circulation

Brain cholesterol is primarily present in the astrocytes and glial cells [50]. In the adult brain, cholesterol is primarily produced by glial cells and taken up by neurons. Astrocytes synthesize cholesterol which is transported with the assistance of ApoE via ATP-binding cassette (ABCA1) to neurons. Cholesterol in the neurons is metabolized to 24S-hydroxycholesterol (24S-OH), which is transported to astrocytes and inhibits cholesterol biosynthesis. Some of 24S-OH are regulated by LXRs and excreted via ABCA1 to synthesize cholesterol in the neurons. Though, another part of 24S-OH is eliminated the systemic circulation [50, 51] (Fig. 3). Cholesterol biosynthesis in the brain is controlled by HMG-CoA reductase, which is a rate-limiting enzyme in the synthesis of cholesterol [47]. Brain cholesterol has a long half-life of up to five years compared to days of peripheral cholesterol [52, 53]. Brain cholesterol is metabolized to oxysterol by a 24-hydroxylase enzyme which is extremely expressed by neurons [54]. Oxysterol can pass into the systemic circulation and excrete by urine which mirrors the rate of brain cholesterol metabolism [54]. Neuronal cholesterol via ABC transporters is excreted to the adjacent neurons [32]. Excreted neuronal cholesterol binds ApoA-I in the cerebrospinal fluid (CSF) and via SR-B1 passes into the systemic circulation [55]. Astrocytes are intricate in synthesizing and releasing LPs in the brains [56], which can pass to the CSF [56]. Dissimilar brain enzymes counting phospholipid transfer protein, cholesteryl ester transfer protein (CETP), and lecithin-cholesterol acyltransferase (LCAT) are involved in the maturation of brain LPs [19]. There are many types of LPs in the brain, though ApoA and ApoE are the main types in the brain [57]. Brain LPs form HDL-like particles and play a role in regulating membrane cholesterol of neurons [58]. Astrocytes, microglia, and oligodendrocytes synthesize ApoE during neuronal injury; it plays an important role in lipid transport between glial cells and neurons [23]. However, ApoA is not formed by the brain but it is transported from circulating HDL via SR-B1 [59]. ApoE acts as a ligand for LDL-related protein 1 (LRP1) and LDLR for cholesterol transport [60].

Furthermore, LXRs are important in regulating brain cholesterol [23]. LXRs are exceedingly expressed in different tissues including the brain; they are activated by oxysterols, mainly 24S-hydroxycholesterol [61]. LXRs augment the expression of ATP-binding cassette transporters, including ABCG1 and ABCA1, which mediate the efflux of cholesterol and phospholipids from astrocytes [55]. LXR agonists improve cholesterol efflux from astrocytes to neurons [56]. LXRs have been shown to modulate brain cholesterol homeostasis at various stages. The neuronal cholesterol concentrations have, thus, been demonstrated to be modulated at three levels; cholesterol uptake by neurons is negatively regulated by LXRs, via the degradation of the LDL receptor by an inducible degrader of LDLR (IDOL). LXR activation by a synthetic agonist stimulated neuronal cholesterol efflux, and LXRs control the cholesterol supply from astrocytes to neurons. The latter pathway is the main source of cholesterol for neurons [23]. Thus, both 24S-OH and GW683965A (a synthetic LXR-agonist) upregulate ABCA1 and ABCG1 in astrocytes, which promotes cholesterol efflux from this cell type. APOE expression is also increased, which mediates the cholesterol transport toward neurons. In oligodendrocytes and Schwann cells, which provide cholesterol to the myelin sheath, LXR can control both cholesterol homeostasis and myelination processes [23, 55, 62]. These findings exemplified that the brain has sole cholesterol metabolism varied from that of circulating cholesterol. Likewise, there is an important interaction between astrocytes and neurons in ruling brain cholesterol metabolism (Fig. 3).

## LXRs and Neurodegeneration

Cholesterol has a key role in synaptogenesis and neurotransmitter release; thus, defective brain cholesterol metabolism is linked with the progression of neurodegenerative disorders [63]. Brain cholesterol homeostasis is maintained by in situ cholesterol biosynthesis and conversion to 24S-OH which passes through BBB into the systemic circulation [64]. Higher circulating 24S-OH level is increased in AD, suggesting brain cholesterol’s role in the pathogenesis of neurodegenerative diseases [64]. Dysregulation of brain cholesterol metabolism induces the expression of the pro-inflammatory renin-angiotensin system (RAS) [65]. Hypercholesterolemia increases 27S-OH levels, a peripheral cholesterol metabolite that can cross BBB and promote the expression of brain RAS in AD patients [65]. These verdicts proposed the association between cholesterol dyshomeostasis and the pathogenesis of neurodegenerative disorders.

LXRs are highly expressed in the adult brain, regulate cholesterol homeostasis, and act as endogenous sensors for intracellular cholesterol [19]. In vitro study demonstrated that LXR agonists improve neuronal differentiation [66]. LXRs have neuroprotective effects against the development of neuroinflammation in different neurodegenerative diseases by inhibiting the expression of pro-inflammatory cytokines [66]. The anti-inflammatory effect of LXRs is mediated by the expression of ubiquitin-like modifier proteins, which inhibit the expression of pro-inflammatory cytokines [67], suggesting the indirect anti-inflammatory effect of LXRs. LXRs promote neurogenesis of midbrain and dopaminergic neurons [66], so LXR agonists may be an effective therapeutic strategy against neurodegenerative disorders, including PD.

Of note, LXRs improve cholesterol efflux, transport, absorption, excretion, and gene expression in astrocytes but not in neurons according to the findings from in vitro study [68, 69]. LXR agonists reduce senile plaque formation by increasing Aβ clearance [69]. However, loss of LXRs in mice triggers the development and progression of neurodegeneration by inducing dysregulation of cholesterol metabolism and age-mediated neuropathological changes [70] suggesting a neuroprotective role of these receptors. Deficiency of LXRs in experimental mice leads to hypoxia, mainly in the SN. Hypoxia and blood vessel changes due to the depletion of LXRs induce neuropathological changes and microvascular dysfunction, a risk factor involved in neurodegeneration [71]. An experimental study revealed that LXR agonists attenuate brain injury in ischemic stroke [72].

Mouzat et al. [73] illustrated that LXRs play a neuroprotective role against the development of amyotrophic lateral sclerosis (ALS) by inhibiting neuroinflammation and promoting the survival of motor neurons. LXR agonist GW3965 attenuates neuroinflammation and regulates brain cholesterol metabolism in AD by increasing ApoE expression, inhibiting astrogliosis, restoration of microvascular morphology, and inhibiting accumulation of Aβ in the blood vessels [71]. Expression of ApoE, which controls cholesterol transport and metabolism, is regulated by LXRs [74]. ApoE is released from astrocytes and acts on neurons expressing LDLR. ApoE regulates and controls cholesterol transport in specific brain regions like SN [75]. Therefore, a defect in ApoE expression induces abnormal cholesterol homeostasis and the development of neurodegeneration. In ApoE knockout mice, lipid droplets accumulate in astrocytes of SN and globus pallidus [76]. Lipid droplets participate in various cellular functions including cell signaling, inflammation, and the development of metabolic diseases. However, the presence of lipid droplets in the CNS is linked with the development of neurodegeneration [76].

In addition, LXR agonists trigger the expression of genes involved in activating cholesterol efflux [77]. As well, ABC transporters reduce cholesterol accumulation in the astroglial cells mainly perivascular astrocytes [78]. Of interest, astrocytes regulate the expression of LDLR in brain endothelial cells and neurons [79]. Therefore, mutations of LXRs disturb BBB permeability causing neuronal injury and the development of neurodegeneration [80]. These findings proposed that LXRs regulate cholesterol brain biosynthesis via control expression of ApoE and ABC transporters.

LXRs are intricate with AD pathogenesis; the experimental study showed that administration of LXR agonist T0901317 reduces deposition of Aβ_1–40_ and Aβ_1–40_ in mice [81]. LXR agonists have been reported to reduce senile plaque formation, increase Aβ clearance, and improve cognitive performance in AD model mice [81]. Inhibition of Aβ by LXR agonists is cell-specific and more neuronal compared to non-neuronal cells [67]. LXRs inhibit the expression of NF-κB and abnormal immune response in AD [82]. In vitro study, LXR agonist GW3965 reduces astrogliosis and improves synaptic plasticity [83]. Endogenous LXR ligands decrease AD-mediated pathology [84], and genetic loss of LXRs in transgenic mice promotes Aβ load. LXRs inhibit the inflammatory response in cultured glial cells to Aβ fibrillary [84]. As well, LXRs improve the phagocytic activity of microglia for Aβ fibrillary [84]. Thus, the signaling of LXRs seems protective against AD’s development and progression. LXRs decrease tau protein phosphorylation in AD patients [85]. The exact and molecular mechanisms of LXRs against AD pathogenesis are not well elucidated. Adighibe et al. [86] revealed that genetic variability of LXRs is associated with an increase in AD risk.

LXR agonist GW3965 can reduce Aβ formation and reverse cognitive deficits in AD model mice by increasing expression of ApoE expression and Aβ clearance [87]. Of interest, the reversal of cognitive deficit occurs by using LXR agonists despite the presence of Aβ in mice [88], suggesting that activation of ABCA1 could be a possible mechanism. In addition, LXR agonists promote Aβ clearance via induction microglia phagocytosis and enzymatic degradation [89]. However, increasing ApoE following LXR agonists may increase PGF2α, which antagonizes the action of LXRs on Aβ clearance and phagocytosis [90]. Moreover, LXR agonists increase cholinergic neurons, synaptic function, and cognitive performance in AD model mice [91]. These findings suggest that LXRs play a critical role against the development and progression of AD.

Furthermore, dysregulation of cholesterol metabolism is linked with the development of ALS [92]. Evidence from clinical findings showed that cholesterol dyshomeostasis is associated with ALS [93]. Increasing CSF cholesterol level and 25-OH cholesterol were shown to be correlated with ALS severity [94, 95]. LXR knockout mice had progressive neuronal loss with a similar phenotype of ALS [96]. LXR receptors are regarded as genetic modulators of ALS through the modulation of energy metabolism [97]. A case-control study involved 438 ALS patients compared to 330 healthy controls showed that genetic variation of ALS genes is associated with 30% of increasing disease severity and duration [97]. Zakyrjanova et al. [98] found that 25-OH cholesterol reduces neuromuscular junction activity by inhibiting LXRs. Therefore, LXRs seem to be protective against the development and progression of ALS.

Furthermore, LXRs and dysregulation of cholesterol homeostasis are associated with the pathogenesis of multiple sclerosis (MS) [99]. LXRs regulate myelination of nerve sheath [100]; thus, defects in LXRs promote the pathogenesis of MS. It has been shown that LXRs are activated by 27S-OH and other oxysterol in MS lesions [101]. A cohort study involving MS patients showed that mRNA of LXRs was increased in peripheral blood mononuclear cells [102] as a compensatory mechanism to counteract immunoinflammatory response. Genetic mutation of LXRs is linked with the development of MS [103], and the use of LXR agonists could be effective in managing MS.

**Fig. 4:**
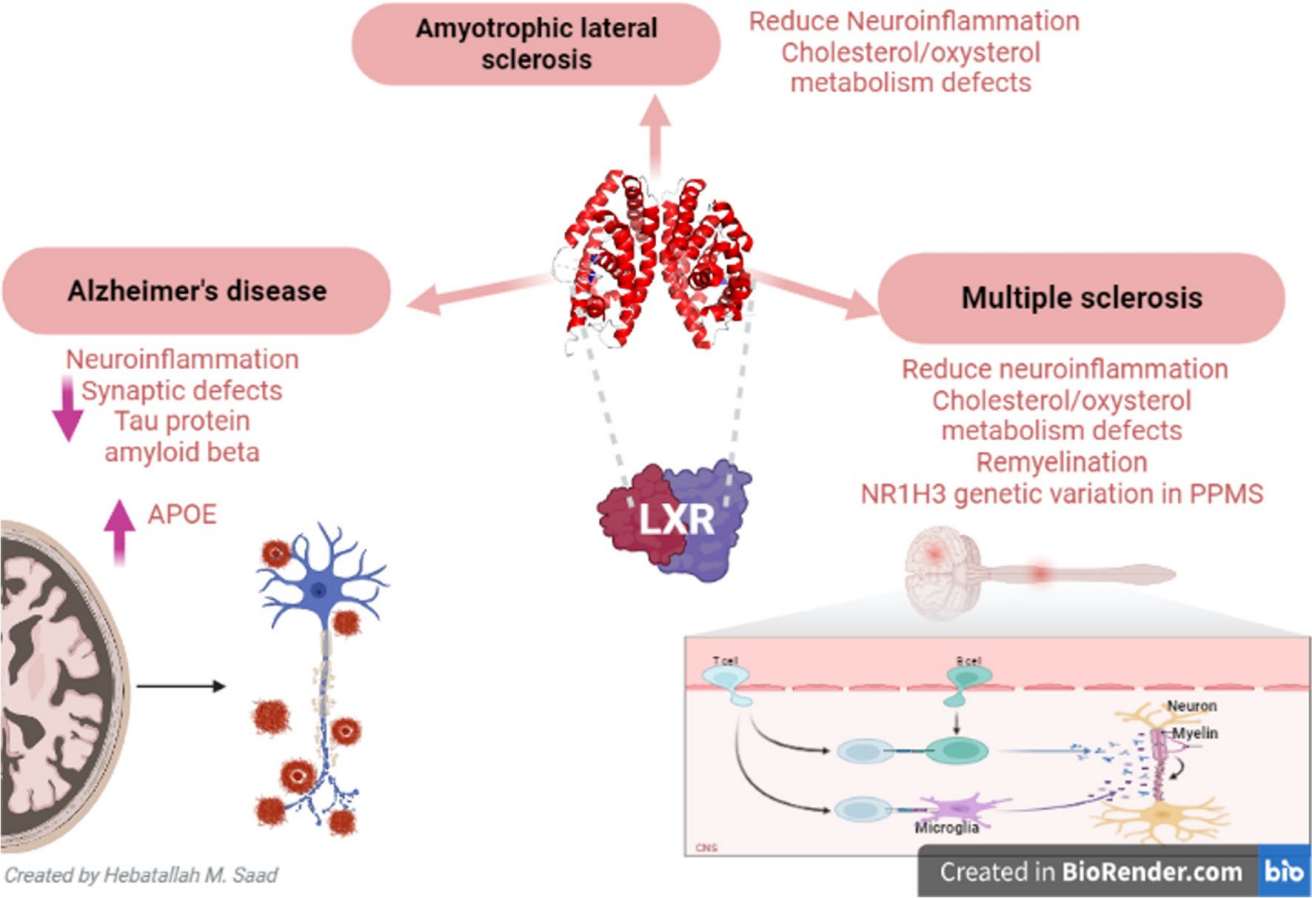
Role of LXRs in neurodegenerative diseases

In sum, LXRs have neuroprotective effects against various types of neurodegenerative disorders and the use of LXR agonists might be effective in this regard (Fig. 4).

## LXRs and PD

It has been shown that LXRβ plays a protective role against the development of PD through the modulation of inflammatory changes in the DNs of the SN [104]. An experimental study demonstrated that LXR agonist GW3965 protects DNs in the SN from the effect of MPTP-induced microglia hyperactivation in mice [104]. LXRβ also promotes the survival of DNs of the SN [105]. Deletion of LXRβ induces the development of PD and other neurodegeneration in mice following six months of age by overactivation of microglia and development of neuroinflammation [106], suggesting a protective role of LXRβ against the development and progression of PD by inhibiting microglia activation.

The deficiency of LXRβ increases vulnerability to the neurotoxic effect of MPTP, and the use of LXRβ agonists reduces astrocyte activation in the SN [104]. LXRβ is highly expressed in DNs and glial cells; therefore, the protection of DNs in experimental PD is not through a direct effect on the DNs but indirectly by inhibiting microglia activation [104]. Therefore, LXRβ agonist attenuates microglia activation-induced neuroinflammation and loss of DNs in MPTP-induced PD [84]. LXRβ agonist TO901317 reduces inflammatory markers and improves mouse locomotor function in MPTP-induced PD by reducing neuroinflammation [107]. As well, LXRβ agonist prevents activation of the pro-apoptotic pathway and development of DNs apoptosis in MPTP-induced PD in mice [108]. However, Marwarha et al. [109] illustrated that LXRβ agonist increases α-synuclein expression. LXRβ promotes midbrain neurogenesis, and activation of LXRβ by oxysterol improves the differentiation of DNs in the SN [66]. Oxysterols like 24S-OH plasma levels are reduced in PD patients; however, a cohort study showed normal 24S-OH plasma levels in PD patients [64]. Both 24S-OH and 27S-OH were increased in the CSF correlated with duration and PD severity [64].

Abnormal brain cholesterol homeostasis promotes aggregation of α-synuclein, leading to cell membrane disruption and DNs loss. Besides, α-synuclein promotes neuronal cholesterol efflux [110, 111]. Furthermore, ApoE enhances α-synuclein aggregation causing cognitive impairment in mice [112]. Therefore, LXRs, through modulation of ApoE and brain cholesterol homeostasis, may lead to controversies regarding their effects on PD. These findings indicated that LXRs have a neuroprotective effect against PD neuropathology. However, the underlying mechanisms of LXRs in PD are not fully elucidated.

## Mechanistic Role of LXRs in PD

### LXRs and Inflammatory Signaling Pathways in PD

#### NF-κB

NF-κB is a DNA-binding protein essential for the transcription of chemokines and pro-inflammatory cytokines. NF-κB is inhibited by an inhibitor of κB (IκB) which sequester NF-κB in the cytosol and prevent its localization [113]. Though, cytokines inhibit IκB with subsequent activation of NF-κB and promulgation of inflammatory disorders [114, 115]. It has been shown that NF-κB intricate in the pathogenesis of PD via induction of inflammation-mediated degeneration of DNs in the SN [116]. Notably, immune dysregulation promotes activation of NF-κB with consequent neuronal injury, neuroinflammation, and development of PD [116]. Findings from postmortem studies revealed a potential role of NF-κB in the degeneration of DNs in the SN. Activation of NF-κB with induction of neuronal apoptosis was established in PD patients compared to the controls [117]. Selective inhibition of NF-κB prevents degeneration of DNs in the SN in a mouse model of PD [118]. Likewise, targeting of NF-κB pathway in the murine PD model may avert PD progression [119]. Different drugs and herbals like pioglitazone, salmeterol, and curcumin delay the degeneration of DNs in the SN by inhibiting NF-κB which is concerned with the progression of neuroinflammation in PD [119]. As well, α-synuclein released from injured DNs triggers activation of NF-κB and release of pro-inflammatory cytokines in a positive-loop manner [120]. These findings proposed that NF-κB could be a therapeutic target in the management of PD. Peculiarly, the Aβ_1–42_ level in the CSF is reduced and not correlated with motor dysfunction in PD patients compared to the controls [121]. In addition, the Aβ_1–42_ level in the CSF is augmented and interrelated with the severity of PD [122]. Nevertheless, Aβ_1–42_ inhibits BBB P-glycoprotein via induction of NF-κB with clearance of Aβ_1–42_ [123]. Consequently, NF-κB not only induces DNs degeneration in the SN but also increases the PD severity through the accumulation of Aβ_1–42_ and α-synuclein.

**Fig. 5:**
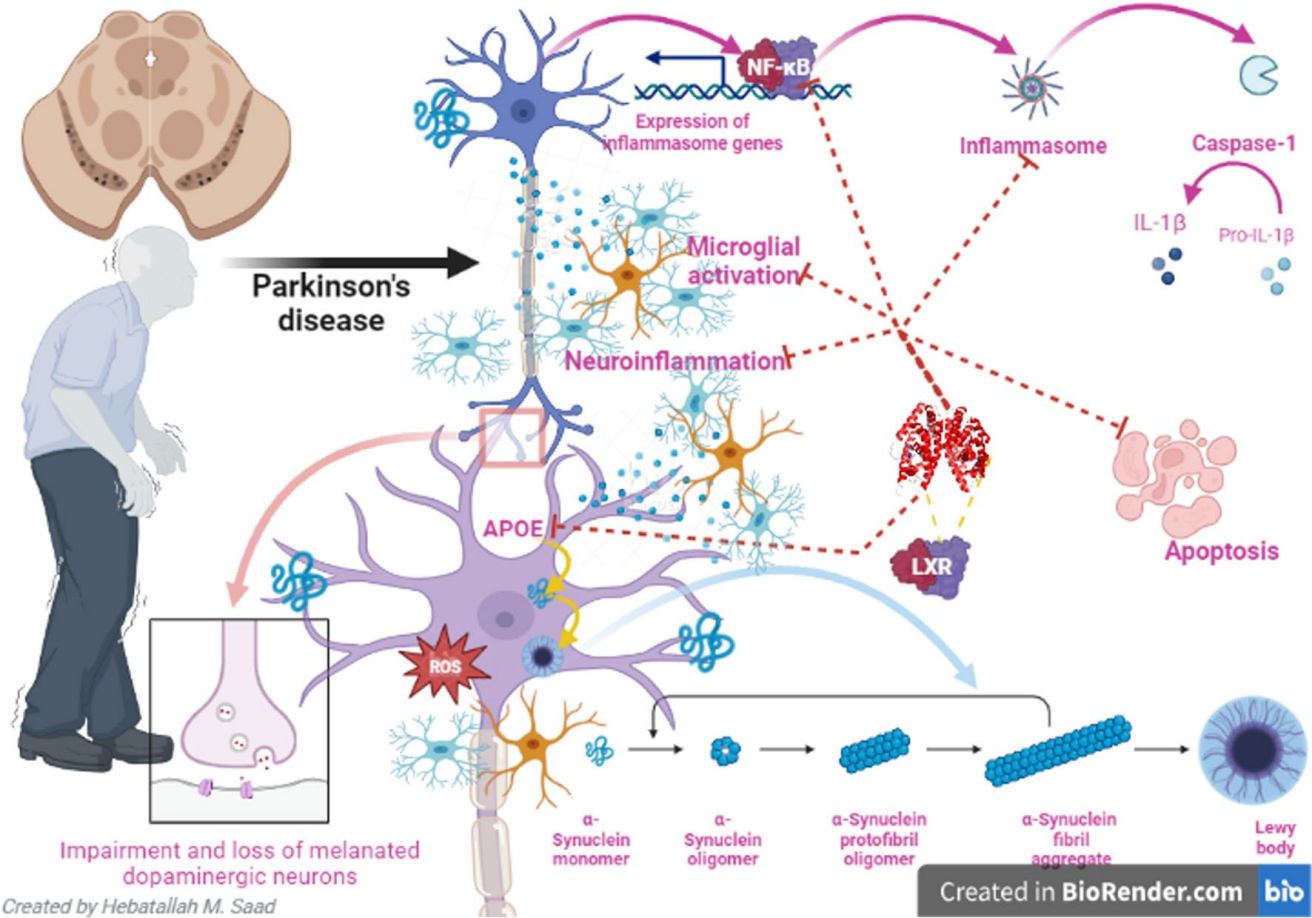
Role of LXRs in Parkinson’s disease neuropathology

LXRs had been reported to inhibit neuroinflammation in PD by reducing the expression of NF-κB [107]. Notably, NF-κB mediates the inhibitory effects of IL-1β on the ABCA1 expression with subsequent alteration of brain cholesterol homeostasis [124]. Lei et al. [125] showed that LXR agonists inhibit the expression of NF-κB in the retinal inflammatory response. In vitro study demonstrated that LXR agonists attenuate LPS-induced IL-8 production and NF-κB activation [126]. In addition, LXR agonists reverse NF-κB by improving IκBα [126]. Of interest, activating LXRs prevents cognitive dysfunction through the modulation of hippocampal synaptic plasticity and macrophage polarization by inhibiting the expression of NF-κB [127]. These observations suggest that LXRs through inhibition of the NF-κB signaling pathway prevent the progression of PD and associated neuroinflammation (Fig. 5).

#### NLRP3 Inflammasome

NLRP3 inflammasome is the nucleotide-binding domain and the leucine-rich repeat-containing family, and pyrin family can form a multiprotein complex. The chief function of NLRP3 inflammasome is the activation of caspase-1 and the maturation of IL-1β and IL-18 [106]. NLRP3 inflammasome is activated by different stimuli counting alternative and non-canonical pathways [128]. NLRP3 inflammasome is activated by NF-κB and sphingosine-1 phosphate [129].

NLRP3 inflammasome is involved in the pathogenesis of PD [130]. NLRP3 inflammasome induces the release of pro-inflammatory cytokines and the progress of neuroinflammation and degeneration of DNs by induction of pyroptosis [130, 131]. In addition, accumulation of the α-synuclein stimulates activation of the microglia with subsequent expression of NLRP3 inflammasome in the SN [130]. Furthermore, systemic activation of NLRP3 inflammasome encourages the accumulation of α-synuclein and degeneration of dopaminergic neurons in the SN [132]. A case-control study included 67 PD patients compared to 24 healthy controls and exhibited that plasma levels of α-synuclein, NLRP3 inflammasome, caspase-1, and IL-1β were increased in PD patients compared to healthy controls [132]. Thus, α-synuclein, NLRP3 inflammasome, and IL-1β plasma levels could serve as biomarkers to screen PD severity and progression. Diverse studies revealed that higher levels of pro-inflammatory cytokines in the CSF and plasma sustenance the interface between the brain and immune system with the progress of neuroinflammation and degeneration of DNs in PD [133, 134]. IL-1β plasma level, a main component of NLRP3 inflammasome, is increased in PD patients [135]. These clarifications anticipated that systemic inflammation via induction of neuroinflammation might lead to the degeneration of DNs and the development of PD. Moreover, increasing of α-synuclein plasma level which is a major constituent of Lewy bodies had been reported to be increased in PD patients compared to the healthy controls [136]. In turn, α-synuclein can activate NLRP3 inflammasome with subsequent release of IL-1β with the progress of systemic inflammation and neuroinflammation [137].

Different studies reported that LXRs inhibit the expression and activation of NLRP3 inflammasome [125, 138]. Activation of LXRs by ginsenosides from *Panax ginseng* reduces NLRP3 inflammasome-induced liver apoptosis in mice [138]. LXR agonist TO90 downregulates NLRP3 inflammasome and linked activation of IL-1β and caspase-1 in mice [125]. LXR agonists attenuate NLRP3 inflammasome-induced peritonitis in experimental mice [139]. Hu et al. [140] observed that LXR agonists reduce inflammatory disorders in different diseases by inhibiting the expression of NLRP3 inflammasome (Fig. 5).

### Neuroinflammation

Neuroinflammation is a process related to the onset of several neurodegenerative disorders, and it is an essential contributor to the pathogenesis and progression of PD [141]. Several damage signals appear to induce neuroinflammation, such as infection, oxidative agents, redox iron, and oligomers of misfolded proteins [142]. Neuroinflammation is responsible for an abnormal secretion of pro-inflammatory cytokines that trigger signaling pathways that activate PD neuropathology [141, 142]. Evidence exists that PD pathogenesis is not restricted to the neuronal compartment but includes interactions with immunological cells in the brain, such as astrocytes, microglia, and infiltrating immune cells from the periphery, which could contribute to the modification of the process of neuroinflammation in PD [143]. Increased BBB permeability and neurovascular dysfunction have been associated with severe conditions in PD [144]. This effect could be associated with infiltrating inflammation molecules to the middle brain, microglia activation, and death of DNs [144]. The systemic inflammatory response in PD seems to be promoted by peripheral lymphocyte activation and augmented levels of pro-inflammatory cytokines [145].

Moreover, neuroinflammation plays an important key role in the pathogenesis of PD. For example, some pro-inflammatory cytokines, such as IL-1β, tumor necrosis factor (TNF)-α, and others, can be found at higher levels in cerebrospinal fluid samples of patients affect with PD compared to age-matched controls [146]. Further supporting the involvement of inflammation, activated microglia can be detected in the brains from living PD patients and post-mortem samples from people affected by PD [146].

It has been shown that activation of LXRs attenuates the development and progression of PD [107]. *In vivo* model of PD using the neurotoxin MPTP revealed that TO901317 administration reduces all of the inflammatory markers intricate in PD such as iNOS and COX2, IκB-α, and NF-κB [147]. Consequently, LXR agonists induce transcriptional activity of LXR target genes, attenuating the astrogliosis and microgliosis induced by neuroinflammation and are widely used in different neurodegeneration animal models [147]. Therefore, TO901317, LXR synthetic agonist, could be a new target in PD [107]. Furthermore, administration of TO901317 prevents the death of DNs by decreasing pro-apoptotic protein which is important in apoptosis [108]. Pre-treatment with TO901317 significantly reduced NF-κB p65 and prevented IκBα degradation in SH-SY5Y in vitro model [107]. Taken together, LXR agonist can modulate the neuroinflammatory pathway involved in PD and can also ameliorate motor function. Therefore, LXR agonists could be studied as a possible pharmacological target in PD.

### Endoplasmic Reticulum Stress and LXR

It has been revealed that endoplasmic reticulum (ER) stress and unfolded protein response (UPR) were linked with PD neuropathology [148]. ER controls the quality of proteins and maintains protein homeostasis via modulating intracellular calcium levels and the folding of proteins synthesized in the cells. The buildup of misfolded proteins in the ER lumen triggers ER stress’ progress with the activation of UPR as a compensatory mechanism to improve the degradation of the misfolded protein [148]. However, in severe ER stress, the activated cellular signaling leads to advanced neuronal injury and the development of PD [13]. Likewise, ER stress induces intracellular Ca^2+^ homeostasis dysregulation by stimulating inflammasomes and autophagy [149]. These verdicts implicate ER stress in the development and progression of PD neuropathology. In the experimental PD model, neurotoxins such as MPTP and 6-hydroxydopamine (6-OHDA) induce the degeneration of DNs through induction ER stress [150]. Brain endogenous morphine biosynthesis was reported to be impaired in PD patients, and exogenous morphine attenuated 6-OHDA-induced cell death *in vitro*. However, the mechanisms underlying neuroprotection of morphine in PD are still unclear [151]. Morphine attenuated the 6-OHDA-induced ER stress in SH-SY5Y cells [151]. Of note, the LXR and lipid-sensor pathways represent a research avenue to identify targets to prevent debilitating complications affecting the peripheral nervous system in obesity [152]. Treatment with LXR agonist GW3965 decreased the mRNA levels of ER stress markers in palmitate-treated dorsal root ganglion explants [152]. A preclinical study revealed that LXR agonist protects DNs in the SN in a mouse model of PD by reducing ER stress [104]. Furthermore, endogenous LXR ligands promote neurogenesis and survival of DNs by inhibiting ER stress [153]. Therefore, ER stress is highly intricate with PD neuropathology, and inhibition of ER stress by LXR agonists may reduce the severity of PD.

### Oxidative Stress, Mitochondrial Dysfunction, and LXRs

Oxidative stress has been reported to play a critical role in PD neuropathology [154]. At the cellular level, PD is linked to surplus production of ROS due to changes in catecholamine metabolism, alteration in mitochondrial electron transporter chain (METC) function, and enhancement of iron deposition in the SN [154]. The failures of normal cellular processes that occur in relation to the aging process are also supposed to add to the increased susceptibility of DNs [155]. Oxidative stress is the fundamental mechanism leading to cellular dysfunction and ultimate cell death. ROS are constantly produced *in vivo* by all body tissues, though oxidative stress occurs when there is an imbalance between ROS production and cellular antioxidant activity. ROS can affect mitochondrial DNA, which can cause modulations in the synthesis of METC components like adenosine triphosphate (ATP) production as well as the leakage of ROS into the cell’s cytoplasm. Selective degeneration of the DNs of the SN may be a source of oxidative stress. Also, the auto-oxidation of dopamine produces electron-deficient dopamine quinones which modify a number of PD-related proteins, such as α-synuclein and parkin [156]. Dopamine quinones can be oxidized to aminochrome, whose redox-cycling leads to the generation of the superoxide radical and the depletion of cellular NADPH [156]. This oxidative process alters mitochondrial respiration and induces a change in the permeability transition pores in brain mitochondria. Mitochondrial dysfunction is closely related to increased ROS formation in PD [157]. Oxidative phosphorylation is the main mechanism providing energy to power neural activity in which the mitochondria use their structure, enzymes, and energy released by the oxidation of nutrients to form ATP. Consequently, this metabolic pathway is the main source of superoxide and hydrogen peroxide, which, at the same time, lead to the propagation of free radicals contributing to PD [157].

Various studies highlighted that LXRs attenuate the development and progression of oxidative stress [158, 159]. Genetic ablation of both LXR isoforms in mice provokes significant locomotor defects correlated with enhanced anion superoxide production, lipid oxidization, and protein carbonylation in the sciatic nerves [158]. Exposure of H9c2 cells to high glucose alone not only caused a significant increase in apoptosis and ROS generation but also led to a decrease in mitochondrial membrane potential, release of cytochrome c, decrease in Bcl-2, increase in Bax expression, and the activation of caspase-3, caspase-9, poly (ADP-ribose) polymerase (PARP), and nuclear factor (NF)-κB. However, pre-treatment with T0901317, a potent agonist of LXR, effectively decreased apoptosis and reduced the levels of ROS [159]. In vitro study demonstrated that oxidative stress downregulates the expression of LXR [160].

Furthermore, LXRs attenuate PD and other neurodegenerative disorders by regulating brain cholesterol metabolism and generation of ROS [161]. Likewise, LXRβ agonists protect DNs in mouse PD model by regulating mitochondrial dysfunction in microglia [104]. In addition, the regulation of mitochondrial dysfunction by LXRs maintains normal brain cholesterol homeostasis [162]. These findings suggest that LXRs play an important role in preventing mitochondrial dysfunction and oxidative stress, which are involved in the pathogenesis of PD.

### Brain-Derived Neurotrophic Factor and LXRs

Brain-derived neurotrophic factor (BDNF) belongs to neurotrophins, a family of proteins that support the function of CNS. BDNF is synthesized mainly in CNS and non-neuronal peripheral cells such as T and B lymphocytes, monocytes, vascular endothelial, smooth, and skeletal muscle cells [163]. BDNF expression was confirmed in the hippocampus, frontal cortex, midbrain, amygdala, hypothalamus, striatum, pons, and medulla oblongata [164]. BDNF plays a key role in the development of the nervous system by affecting cell differentiation, neuronal development, growth and survival, neurogenesis, synaptogenesis, and synaptic plasticity [163, 164]. The pre-proBDNF precursor is synthesized in the endoplasmic reticulum and then transported to the Golgi apparatus, where the preregion sequence is cleaved to produce the proBDNF isoform. Then, proBDNF may be converted into mature BDNF in the trans-Golgi network by the subtilisin-kexin family of endoproteases such as furin or in intracellular vesicles by convertases [165]. ProBDNF and BDNF exert their biological activity by binding to two types of cell surface receptors, the Trk tyrosine kinases, and the p75 neurotrophin receptor (p75NTR) [166]. The neuroprotective effect of BDNF results from activation of the TrkB pathway, which leads to attenuation of apoptosis, glutamate, and nitric oxide (NO) neurotoxicity and cell damage caused by oxidative stress. An increase in oxidative stress, glutamate neurotoxicity, NO production, and the process of apoptosis are observed in PD [167, 168]. Preclinical findings revealed that BDNF expression was reduced in animal model of PD [169, 170]. A case control study that included 47 PD patients and 23 healthy controls revealed that BDNF serum level was reduced significantly in the early stage of PD patients compared to controls [171]. Later on, with the progression of PD severity, BDNF serum level was increased and correlated with disease severity [171]. It has been shown that LXRs promote ventral midbrain neurogenesis in vivo and in human embryonic stem cells by increasing the expression of BDNF [66]. As well, BDNF promotes cholesterol biosynthesis and encourages the accumulation of presynaptic proteins in cholesterol-rich lipid rafts by increasing of expression of LXRβ [172]. This finding indicated that BDNF plays a critical role in the modulation of cholesterol homeostasis in glial and neuronal cells through LXR-dependent pathway. Furthermore, preclinical study demonstrated that LXR agonist promotes BDNF expression in the neurons [173]. These observations suggest that LXR agonists through modulation of BDNF expression could be effective in managing PD.

Therefore, inhibition of NLRP3 inflammasome and NF-κB by LXRs could effectively prevent neuroinflammation in PD. Taken together, LXRs play a crucial role in PD neuropathology by reducing the expression of inflammatory signaling pathways, neuroinflammation, oxidative stress, mitochondrial dysfunction, and enhancement of BDNF signaling. However, the present study had many limitations, including a paucity of clinical studies and most of the current findings obtained from preclinical studies that correspond not merely human applications. Thus, clinical trials to determine the effects of LXR agonists on PD neuropathology are recommended in this regard (Table 1).

**Table 1:** The effects of liver X receptors (LXRs) agonists on Parkinson’s disease (PD) neuropathology

Study type	Findings	Ref.
Experimental studies	TO901317, LXR synthetic agonist reduces the expression of NF-κβ in the PD mouse model. Pre-treatment with TO901317 significantly reduced NF-κB p65 and prevented IκBα degradation in SH-SY5Y in vitro model.	Paterniti et al. [107]
Experimental study	LXR agonists attenuate NLRP3 inflammasome-induced peritonitis in experimental mice.	Yu et al. [139]
Experimental study	LXR agonist TO90 downregulates NLRP3 inflammasome and linked activation of IL-1β and caspase-1 in mice.	Lei et al. [125]
Experimental study	LXR agonists induce the transcriptional activity of LXR target genes and attenuate neuroinflammation in mice.	Riddell et al. [147]
In vitro study	LXR agonist GW3965 decreased the ER stress markers in palmitate-treated dorsal root ganglion explants.	Gavini et al. [152]
In vitro study	LXR ligands promote neurogenesis and survival of DNs by inhibiting ER stress.	Theofilopoulos et al. [153]
In vitro study	Pre-treatment with T0901317, a potent agonist of LXR, effectively decreased apoptosis and reduced the levels of ROS.	Cheng et al. [159]
Experimental study	Likewise, LXRβ agonists protect DNs in mouse PD models by regulating mitochondrial dysfunction in microglia.	Dai et al. [104]
In vitro study	LXRs promote neurogenesis in vivo and human embryonic stem cells by increasing the expression of BDNF.	Sacchetti et al. [66]

## Conclusions

PD is the second most common neurodegenerative disease due to the progressive degeneration of DNs in the SN. LXRs are the most common nuclear receptors of transcription factors that control cholesterol metabolism and have pleiotropic effects, including anti-inflammatory effects, reduced intracellular cholesterol accumulation, immune regulation, anti-proliferative effects, anti-tumor effects, and prevention development of endoplasmic reticulum stress. LXRs have neuroprotective effects against the development of neuroinflammation in different neurodegenerative diseases. LXRs regulate cholesterol brain biosynthesis via control expression of ApoE and ABC transporters. Inhibition of NLRP3 inflammasome and NF-κB by LXRs could effectively prevent neuroinflammation in PD. Taken together, LXRs play a crucial role in PD neuropathology by inhibiting neuroinflammation and associated degeneration of DNs. Therefore, clinical trials to determine the effects of LXRs agonists on PD neuropathology are suggested in this regard.

## Data Availability

Not applicable.
